# RETROSPECTIVE ANALYSIS OF PATIENTS WITH IMMEDIATE DECANNULATION IN SEVERE ACQUIRED BRAIN INJURY (RAPID-SABI)

**DOI:** 10.2340/jrm-cc.v8.42068

**Published:** 2025-02-05

**Authors:** Robbert-Jan VAN HOOFF, Mette LINDELOF, Emma GHAZIANI, Trine HØRMANN THOMSEN, Christina KRUUSE, Christian GUNGE RIBERHOLT, Charlotte RATH

**Affiliations:** 1 Department of Brain and Spinal Cord Injury, Copenhagen University Hospital – Rigshospitalet, Copenhagen, Denmark; 2 Department of Neurology, Neurovascular Research Unit, Copenhagen University Hospital – Herlev and Gentofte, Copenhagen, Denmark

**Keywords:** severe acquired brain injury, tracheostomy, decannulation

## Abstract

**Objective:**

To examine an early decannulation protocol in adult severe acquired brain injury (SABI) patients.

**Design:**

Retrospective, observational cohort study.

**Subjects/patients:**

Tracheotomized SABI patients ≥ 18 years admitted to a neurorehabilitation unit.

**Methods:**

Primary outcome measure was difference in survival rate within first year of discharge. Secondary outcome measures were respiratory infections treated with antibiotics, rate of re-cannulation, time from admission to decannulation, length of stay, difference in rate of re-admission due to pneumonia within first year of discharge and difference in rate of tracheal tube dependency within first year of discharge.

**Results:**

No statistical significance in survival rate within the first 12 months from discharge was found. Median time from admission to decannulation was 32 days (interquartile range [IQR] 14–61) vs 9 days (IQR 0–13) in the control and intervention group, respectively (*p* < 0.0003). Median length of stay was 66 days (IQR 54–92) in the control group vs 60 (IQR 48–75) days in the intervention group (*p* = 0.168).

**Conclusion:**

A new early decannulation protocol omitting evaluation of tolerance to tracheostomy tube capping and fiberoptic endoscopic evaluation of swallowing was non-inferior to previous procedures in survival rate within first year of discharge. The early decannulation protocol allowed for significantly earlier decannulation.

## LAY ABSTRACT

This study aimed to examine what the influence of earlier removal of a tracheostomy is on the mortality within the first year after discharge in patients with severe acquired brain injury. One group of 27 patients that was treated under the new protocol was compared to a group of 34 patients that was treated with the previous protocol. All adult patients admitted to our neurorehabilitation unit with a tracheostomy were included. Our results showed that the new protocol did not increase the risk of mortality within the first year of discharge from our unit. However, the tracheostomy could be removed earlier in patients treated under the new protocol. Moreover, our study showed a tendency to a shorter stay in our neurorehabilitation unit for patients who were treated with earlier removal of tracheostomy. The new protocol should be investigated in prospective studies to confirm our findings.

Tracheostomy may cause several complications, which increase with prolonged permanence of the tube (1). The complications, such as bleeding, pneumonia, tracheal stenosis, dehiscence, and granuloma formation (2–4), delay, and complicate subsequent rehabilitation. After severe acquired brain injury (SABI), which include severe traumatic brain injury, stroke, anoxic brain damage, 21 to 47% of patients are tracheostomized in the acute phase (5). The choice of tracheostomy is based on the patient’s prolonged inability to breathe and/or protect their airway sufficiently and is usually done in the intensive care unit (ICU). Decision on the removal of a tracheostomy is an interdisciplinary evaluation of the clinical condition with assessment of good secretion management and reactive coughing, which are considered key factors for successful decannulation (6–9). Wavering decannulation associated with longer hospital stay and increased healthcare costs (10). Moreover, having a tracheostomy can be a factor slowing down rehabilitation goals (2–3) such as vocal and swallowing recovery (11). One observational study (12) found the presence of airway lesions in 67% of patients with long-term tracheostomy after SABI. Therefore, decannulation is of high importance and should be considered a main early rehabilitative goal. Several extensive decannulation protocols have been proposed (13). Such protocols, however, may include criteria’s that are difficult to assess or require special equipment not readily available, which can prolong time to decannulation. The variability in decannulation protocols reflects an evidence-gap on when and how to decannulate a patient with SABI for optimal outcome (14).

We aimed to retrospectively evaluate safety and clinical outcome after implementation of a new decision protocol with early evaluation and decannulation of tracheostomized SABI patients after admission to a specialized Neurorehabilitation Unit (NU). We hypothesized that implementing an early decannulation protocol in adult patients with SABI would not result in increased mortality and would improve safety outcomes, as evidenced by reduced complications associated with the new decannulation protocol.

## METHODS

### Data collection

Data were extracted from the patient’s electronic medical records by 2 authors (RH and CR) and transferred to a predefined excel sheet. The study was approved by the Capital Region of Copenhagen (p-2023-14366). No further ethical approval was needed for this retrospective study according to Danish law. The study was registered at ClinicalTrials.gov (Identifier: NCT06167538).

### Design

In this retrospective, observational cohort study it was decided to assess the novel early decannulation process of SABI patients implemented from the 1st of September 2021 to reduce tracheostomy-related complications.

### Objectives

The primary objective was to evaluate the safety based on differences in rate of survival and clinical parameters of this earlier decannulation protocol compared to the previous protocol in adult patients with SABI at the NU.

### Participants and setting

The Department of Brain and Spinal Cord Injury, Copenhagen University Hospital Rigshospitalet is a specialized NU for SABI patients with highly complex rehabilitation needs. In addition to the specialized neurorehabilitation beds, the department has semi-intensive neurological beds and an expertise in decannulation of tracheostomized patients, in which both mechanical ventilation and weaning have been ceased. The department has a catchment area consisting of Eastern Denmark, the Faroe Islands and Greenland (approximately 2.8 million inhabitants).

All SABI patients ≥ 18 years admitted to the NU from September 2021 to October 2022 with a tracheal cannula, were consecutively screened and included when identified as eligible candidates in the study. However, when deciding to decannulate, the patient was assessed by the multidisciplinary treating team with the final decision made by the treating neurologist. Several factors were considered in the timing and choice of decannulation: patient’s level of consciousness, hemodynamical stability, need for and frequency of salivary aspiration, cough strength, and respiration frequency (Table I). Patients could not be treated for pneumonia and simultaneously have respiratory instability at the time of decannulation. Most importantly, the new early decision protocol did not include test of tracheostomy tube capping (i.e. testing the ability to breathe through mouth and nose) for at least 24 h or longer or assessment of silent aspiration by fiberoptic endoscopic evaluation of swallowing (FEES). The SABI patients with a tracheal tube admitted between July 2019 until December 2020 served as a historical control group in which the former procedure was used (Table I).

**Table I:** Protocol criteria for decannulation

Procedure criteria	Control group (standard procedure) *n* = 34	Intervention group (new procedure) *n* = 27
Tolerance to ≥ 24 h of tracheotomy tube capping	X	
No current pneumonia (assessed and defined by the treating physician based on (para) clinical findings)	X	
Respiration (RF < 25, sat > 92%)		X
Assessment risk of aspiration including cough and spontaneous swallowing	X	
Absence of aspiration in all positions. When in doubt assessment by FEES	X	
Assessment of secretions (need for suctioning, amount, consistency)		X
Minimal or no need for suctioning secretions	X	
Consciousness (general assessment by the treating physician, which might include level of consciousness, GCS and FOUR scores)		X
Vital signs (i.e. blood pressure, heart rate, respiratory rate and oxygen saturation)		X
Absence of reflux/vomit		X
Decannulation performed by	Physician, OT or nurse	Physician

OT: occupational therapist; FEES: fiberoptic endoscopic evaluation of swallowing.

There were no exclusion criteria.

### Primary outcome measure

Difference in rate of survival within the first year of discharge between the old and new protocol in patients with SABI and a tracheal tube admitted to the NU.

### Secondary outcome measures

Respiratory infection was estimated as the difference in the rate of at least 1 instance of antibiotics prescribed to the treatment of pneumonia (as determined by the treating physician based on clinical and/or paraclinical findings such as blood samples, imaging techniques or microbiology) following decannulation, at discharge, 2 months post-discharge and 12 months after symptom onset . The effect on need for re-cannulation was calculated as the difference in the rate of re-cannulation of the tracheal tube after first attempt of decannulation. The time from admission to decannulation was also calculated. The overall impact of complications was estimated as the difference in the length of stay at the NU (measured from admission to the department of Brain and Spinal Cord Injury) for patients admitted with a tracheal tube. Long-term effect on the risk of infection was evaluated as the difference in rate of at least 1 re-admission due to pneumonia at 2 months after discharge and 12 months after ictus. Finally, the success rate of decannulation was estimated as a difference in rate of patients dependent on a tracheal tube at discharge, 2 months after discharge, and 12 months after symptom onset.

### Statistical analysis

Statistical analysis was performed with SPSS version 29.0.1.0. We assumed that the new decannulation procedure would be non-inferior to the former procedure regarding survival rate. Frequency and percentages were used to summarize patients’ baseline data and completion of clinical outcomes. Normally distributed data are presented as mean (±SD), while non-normally distributed data are presented as median (IQR). The mean survival times for both the 3- and 12-month periods were calculated and reported using descriptive statistics. To compare the survival rates and the secondary outcome measures we used an independent samples *t*-test when all assumptions of normal distribution was present. If not, a Wilcoxon 2 sample test (continuous variables) or Fisher’s exact test (categorical variables) was applied to compare the medians between the 2 groups. *P*-values of 0.05 or less were considered significant.

### Data availability

De-identified data not published within this article will be made available by request from any qualified investigator.

## RESULTS

### Patient characteristics

**Fig. 1:**
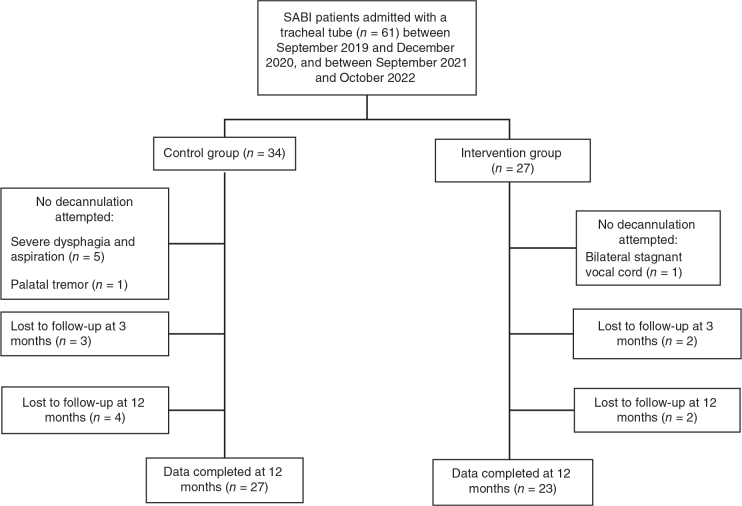
Study flow chart.

In the control group, we identified 34 SABI patients with tracheostomy upon admission, 27 tracheotomized patients were identified in the intervention group. Both groups included more males (61.8 and 59.3%) than female patients. Median age was 58 years (IQR 47–65 years) in the control group, and 49 years (IQR 39–62 years) in the intervention group (*p* = 0.238). Comorbidities are defined and presented by ICD code (Table II). Decannulation was attempted in 28 out of 34 (82.4%) patients in the control group, and in 26 of 27 patients (96.3%) in the intervention group (Fig. 1).

**Table II:** Descriptive and clinical characteristics of the patients in the control and intervention group

Characteristics	Control (*n* = 34)	Intervention (*n* = 27)
**Sex**		
Male (*n*, %)	21 (62)	16 (59)
Female (*n*, %)	13 (38)	11 (41)
**Age** (median, IQR) in years	58 (47–65)	49 (39–62)
**Comorbidities (ICD code)**		
History of hypertension (*n*, %)	8 (24)	8 (30)
Diabetes mellitus type 2 (*n*, %)	2 (6)	3 (11)
Previous ischemic stroke/TIA (*n*, %)	4 (12)	1 (4)
Atrial fibrillation (*n*, %)	1 (3)	1 (4)
Cerebral aneurism (*n*, %)	3 (9)	0
**Diagnosis:**		
Intracerebral hemorrhage (*n*, %)	12 (35)	6 (22)
Traumatic brain injury (*n*, %)	7 (21)	7 (26)
Subarachnoidal hemorrhage (*n*, %)	6 (18)	3 (11)
Acute ischemic stroke (*n*, %)	4 (12)	4 (15)
Subdural hematoma (*n*, %)	0	2 (7)
Cerebral anoxia (*n*, %)	0	2 (7)
Basilar aneurism (*n*, %)	2 (6)	0
Cerebral abscess (*n*, %)	1 (3)	0
Surgery meningioma (*n*, %)	1 (3)	0
AV malformation (*n*, %)	1 (3)	0
Postoperative complications (*n*, %)	0	3 (11)
**Glasgow Coma Score < 10**		
Yes (*n*, %)	22 (65)	19 (70)
No (*n*, %)	9 (26)	7 (26)
Unknown (*n*, %)	3 (9)	1 (4)
**Brainstem lesion**		
Yes (*n*, %)	4 (12)	4 (15)
No (*n*, %)	30 (88)	23 (85)
**Hydrocephalus**		
Yes (*n*, %)	11 (32)	10 (37)
No (*n*, %)	23 (68)	17 (63)
**Space-occupying cerebellar lesion**		
Yes (*n*, %)	5 (15)	4 (15)
No (*n*, %)	29 (85)	23 (85)
**ICH volume >25 mL**		
Yes (*n*, %)	8 (24)	2 (7)
No (*n*, %)	3 (9)	7 (26)
N/A (*n*, %)	22 (65)	18 (67)
Unknown (*n*, %)	1 (3)	N/a
**Ischemia MCA >2/3**		
Yes (*n*, %)	0	0
No (*n*, %)	0	1 (4)
N/a (*n*, %)	34 (100)	26 (96)
**Neurosurgical intervention**		
Yes (*n*, %)	27 (79)	21 (78)
No (*n*, %)	7 (21)	6 (22)
**Sepsis**		
No	34 (100)	34 (100)

ICH: intracerebral hemorrhage; TBI: traumatic brain injury; SAH: subarachnoidal hemorrhage; AIS: acute ischemic stroke; SDH: subdural hematoma; AV: malformation = arteriovenous malformation; MCA: middle cerebral artery.

Assessment of the presence of hydrocephalus and space-occupying cerebellar lesion was based on the initial CT scan’s radiological report.

### Primary outcome

Survival rate at was 91.2% (31 of 34 patients) in the control group, vs 92.6% (25 of 27 patients) in the intervention group (*p* = 1.000). Survival rate at 12 months from discharge was 79.4% (27 of 34 patients) in the control group vs 85.2% (23 of 27 patients) in the intervention group (*p* = 0.526) (Table III).

**Table III:** Primary and secondary outcome measures

Outcome measures	Control group (*n* = 34)	Intervention group (*n* = 27)	*p*
**Primary outcomes**			
Mortality at 3 months after discharge (*n*)*	3	2	1.000
Mortality at 12 months after discharge (*n*)*	7	4	0.526
**Secondary outcomes**			
Re-cannulation (*n* [%])*	1 (3.6)	1 (3.7)	1.000
Time from admission to decannulation (median [IQR])†	32 [14–61]	9 [0–13]	< 0.0003
Length of stay (median [IQR])†	66 [54–92]	60 [48–75]	0.168
Dependency on tracheal tube at 3 months after discharge (*n* [%])*	4 (12.5)	1 (4)	0.362
Dependency on tracheal tube at 12 months after discharge (*n* [%])*	3 (10.7)	1 (4.3)	0.610
At least 1 re-admission due to pneumonia at 2 months (*n*)*	4	0	0.123
At least 1 re-admission due to pneumonia at 12 months (*n*)*	21	10	0.073
At least 1 episode of antibiotic use against pneumonia upon discharge (*n*)*	10	4	0.228
At least 1 episode of antibiotic use against pneumonia at 2 months (*n*)*	19	10	0.198
At least 1 episode of antibiotic use against pneumonia at 12 months (*n*)*	21	10	0.073

* Fisher’s exact test, or

† ,Wilcoxon 2 sample test were used to compare the secondary outcome measures between the 2 groups.

### Secondary outcomes

During admission at the NU, there were 30 episodes of antibiotic use based on clinical suspicion of pneumonia in the control group, while 17 episodes in the intervention group. At 2 months from discharge there had been 12 episodes of antibiotic use for pneumonia in the control group, while there had been 2 episodes in the intervention group. Finally, at 12 months after ictus there had been 2 episodes of antibiotic use against pneumonia in both groups.

Re-cannulation after initial decannulation was required in 1 patient in each group, that is in one of the 27 (4%) patients in the intervention group vs in one of 28 (4%) patients in the control group decannulation were attempted (*p* = 1.000) (Table III). The re-cannulation was in both patients done due to occurrence of pneumonia, which required increased oxygen supply and clearance of secretion by manual suction.

Median time from admission to decannulation was 32 days (IQR 14–61) in the control group vs 9 days (IQR 0–13) in the intervention group (*p* < 0.0003), with 1 patient decannulated 369 days after admission in the latter group.

Median length of stay at the NU was 66 (IQR 54–92) days in the control group vs 60 (IQR 48–75) days in the intervention group (*p* = 0.168).

At 2 months from discharge 4 out of 34 patients (11.8%) were re-admitted at least once due to pneumonia and at 12 months after ictus 21 out of 34 patients (61.8%) had at least 1 re-admission due to pneumonia in the control group. In the intervention group no patients were readmitted due to pneumonia at 2 months after discharge, while 10 out of 27 patients (37.0%) were re-admitted at least once due to pneumonia at 12 months after ictus.

In the control group 4 of the surviving 32 patients (13%) and 3 of the surviving 28 patients (11%) were still dependent on a tracheal tube at 2 months and 12 months after discharge, respectively. In contrast, dependency on a tracheal tube in the intervention group was seen in one of the 25 surviving patients (4%) and one of the 23 surviving patients (4%) at 2 and 12 months after discharge, respectively.

## DISCUSSION

In this retrospective cohort study on patients admitted to a NU, implementation of a new early decannulation protocol omitting previous assessment of tolerance to ≥ 24 h of tracheotomy tube capping and assessment by FEES, did not reduce survival rate. The early decannulation protocol reduced the median time to decannulation from 32 to 9 days without significantly affecting morbidity or need for re-cannulation at 3 or 12 months after discharge compared to the former procedure. Importantly, less patients were readmitted 12 months after ictus in the intervention group and there was a trend towards a decrease in the length of stay after change in the decannulation protocol. It is notable, that the control and intervention group are comparable in terms of relevant characteristics, such as co-morbidities, consciousness at symptom onset and other potential confounding factors.

Good secretion management and reactive coughing are generally considered to be a key factor for successful decannulation (6–9). In a systematic review (13) effective coughing and tolerance of tube capping for at least 24 h were shown to be the most relevant parameters for successful decannulation. However, a later retrospective study (1) identified that the presence of an effective cough and the presence of a spontaneous cough were the factors associated with successful decannulation in SABI patients in a neurorehabilitation ward. Moreover, the authors found that Glasgow Coma Scale (GCS) score, the type of tube used, and relative capping did not show a correlation with successful decannulation. Another retrospective single-center study (14) examining a cohort of prolonged mechanically ventilated, tracheotomized patients found that severe dysphagia and long-term dependence on ventilator were the reasons for decannulation failure (overall failure rate of 41%). However, this cohort did not exclusively include SABI patients. The conflicting results found in the various studies reflect the paucity of evidence on successful decannulation strategies in SABI patients. The results from our study partially confirm previous results (1), assessing that the presence of an effective and spontaneous cough are factors associated to successful decannulation.

A systematic review by Wahlster et al. (5) found a pooled long-term mortality (6–12 months) of 21% for tracheostomized SABI patients in a (neuro)ICU. It is noteworthy that among patients who were admitted with a tracheostomy to a rehabilitation unit, mortality rates ranged between 3 and 10% (5). This is in line with the mortality rates reported in the current study and may reflect a selection-bias of including patients estimated to have a better prognosis to further neurorehabilitation. There was no significant difference in mortality between the 2 decannulation cohorts in this study, which could be due to the small numbers and lack of statistical power.

We found a successful decannulation rate of 96.3% in the intervention group (i.e. 25 out of 26 patients in whom decannulation was attempted). This contrasts with other retrospective studies that found a lower successful decannulation rate (11, 15) (63 and 57%, respectively) compared to the current data, however one (15) included a higher number of patients (90.6%) with comorbid pulmonary conditions than this study (64% of the patients).

A recent Italian study (11), reported a mean time from admission to the rehabilitation unit to decannulation of 59 days contrasting the median 9 days in our intervention group. Reducing time to decannulation may improve both immediate and long-term clinical outcome, since prolonged tracheal tube presence associate to important clinical complications and might slow down neurorehabilitation process. Nevertheless, our retrospective study was not powered to identify potential effects on clinical outcomes of the new decannulation protocol.

This study has several limitations. Firstly, it is a retrospective, observational cohort study and relies on existing data, which may not have included all relevant variables or outcomes of interest. Limited availability of data on certain variables, especially the functional outcome parameters, may restrict the scope and depth of the analysis, which impact the ability to draw firm conclusions and only be hypothesis generating. However, the 2 groups were comparable in terms of specific characteristics and only few percentages of data were missing. Secondly, since data were collected after the outcome had occurred, it may be difficult to ascertain the sequence of events. Also, we pooled SABI patients with different underlying etiologies. One study (1) found a different success rate for decannulation in 3 groups (i.e. stroke, TBI and cerebral anoxia) with the highest success rate in TBI patients. The latter was confirmed by a retrospective multicenter study (16). Furthermore, a higher decannulation rate in SAH patients vs ICH/AIS patients, has been found (5). Therefore, in a larger cohort, it may have been more valid to divide the groups based on their underlying etiologies. However, as this is a single-center study, we were able to include only relatively small cohorts generally limiting its statistical power.

In conclusion, a novel early decannulation protocol for SABI patients omitting assessment of tolerance to tube-capping and FEES, showed a significantly earlier decannulation without affecting survival rate, morbidity, or number of re-cannulations up to 12 months follow-up after discharge. A trend towards a reduced length of stay and less readmissions, was found. Additionally, a reduced use of antibiotics was recorded after the new early decannulation protocol was implemented, but future large, randomized trials should confirm these results.
